# Subretinal Injection of HY Peptides Induces Systemic Antigen-Specific Inhibition of Effector CD4^+^ and CD8^+^ T-Cell Responses

**DOI:** 10.3389/fimmu.2018.00504

**Published:** 2018-03-13

**Authors:** Julie Vendomèle, Safa Dehmani, Quentin Khebizi, Anne Galy, Sylvain Fisson

**Affiliations:** ^1^Généthon, Inserm UMR_S951, Univ Evry, Université Paris Saclay, EPHE, Evry, France

**Keywords:** anterior chamber-associated immune deviation, eye, ocular immune privilege, subretinal-associated immune inhibition, T-cells

## Abstract

**Purpose:**

Injection of an antigen into the anterior chamber of the eye induces a peripheral antigen-specific immune modulation mechanism, known as anterior chamber-associated immune deviation (ACAID). Delayed-type hypersensitivity experiments argue that the subretinal space (SR) of the eye displays properties similar to ACAID. However, no investigation was performed regarding the differential impact of a subretinal antigen injection on peripheral CD4^+^ versus CD8^+^ T cells, on the potential immune deviation regarding Th profiles, and on the antigen-specificity of the inhibition. A better understanding of these mechanisms is crucial to improve safety and immunomonitoring of ongoing therapeutic approaches targeting the SR. The aim of this study is to characterize the proliferative capacities and cytokine patterns of antigen-specific CD4^+^ and CD8^+^ T cells after a subretinal injection of antigen in mice.

**Methods:**

Ubiquitously Transcribed tetratricopeptide repeat gene Y-linked (UTY) and DEAD Box polypeptide 3 Y-linked (DBY) peptides which respectively include MHCI- and MHCII-restricted T-cell epitopes of the mouse HY male antigen, were injected into the subretinal space of C57BL/6 female mice. 2 weeks later, these mice were immunized subcutaneously with these peptides and compared to control mice. A week later, T-cell immune responses were analyzed by IFNγ ELISpot assays and cytokine measurements (IL-2, IL-4, IL-6, IL-10, IL-13, IL-17a, IFNγ, TNFα, GM-CSF, and MCP-1) in the spleen and with proliferation assays in draining lymph nodes.

**Results:**

Immune cells from mice that received HY peptides in the SR before immunization, compared with those from control immunized mice, secreted significantly smaller quantities of Th1/Tc1, Th2/Tc2, and Th17/Tc17 cytokines, and HY-specific CD4^+^ T cells proliferated less in response to HY peptides.

**Conclusion:**

Taken together, our data clearly demonstrate that the subretinal injection of HY peptides induces a systemic HY-specific inhibition of conventional Th profiles and CD8^+^ T cells. We propose to call this phenomenon SRAII, for subretinal-associated immune inhibition.

## Introduction

In recent decades, the eye has been considered an immune-privileged site due to its unique anatomical and immune properties. Physical barriers, such as the blood–retinal barrier, limit and control exchanges between the eye and the rest of the organism. Local immunomodulatory properties are provided by the secretion of immune suppressive factors, such as TGFβ. Moreover, systemic immunomodulatory activities have been reported. Injecting an antigen into the anterior chamber of the eye induces an antigen-specific systemic immune deviation known as anterior chamber-associated immune deviation (ACAID) (1–3). This mechanism was first described as a deviation of CD4^+^ T helper (Th) cell effector function from a Th1 (IL-2, IFNγ, TNFα) toward a Th2 (IL-4, IL-10, IL-13) profile (4, 5). Other studies report that ACAID can inhibit the Th1 profile without affecting the Th2 profile (6, 7) and can repress Th2-mediated pulmonary pathology (8). Another study reports that this immune inhibition is due to the generation of regulatory T cells (Tregs) rather than deviation from a Th1 to Th2 profile (9), although the deep characterization of cytokine signatures (e.g., IL-6 and GM-CSF proinflammatory cytokines, as well as chemoattractant molecules, such as MCP-1), Th profiles (e.g., IL-17 for Th17) and Tc profiles (10) have not yet been performed. The induction of this mechanism in the eye does not appear to be restricted to the intracameral route of antigenic delivery. Delayed-type hypersensitivity (DTH) experiments using ovalbumin showed that the subretinal space (SR) displays a pattern of immune modulation which is similar to ACAID (11). Moreover, the retinal expression of a neo-antigen induces the generation of antigen-specific Treg cells in the periphery (12). These Treg cells can be generated locally, independently of the thymus, and are able to limit autoimmune reactions (13, 14). However, compared to ACAID, there is a lack of deep investigation of Th profiles and CD8^+^ T cells, although the SR is the target of a growing number of emerging treatments, especially for therapies targeting photoreceptors or retinal pigment epithelium. Gene and cell-based therapies have emerged as a promising approach to treat various ophthalmic diseases of genetic or age-related origins. In the past few years, more than 25 clinical trials of gene therapy for genetic and nongenetic diseases (e.g., respectively, Leber’s congenital amaurosis and age-related macular degeneration) and 24 of cell therapy have been initiated (15). At least 30 of these have administered treatment *via* subretinal injections. Although these trials appear to meet safety criteria, the impossibility of immune monitoring of cells other than peripheral blood cells has prevented an in-depth investigation of the immunologic mechanisms involved in these innovative approaches. These therapies, which involve the injection of multicellular or multiprotein components, may well trigger and affect the immune properties of the eye, especially as these treatments are administered in diseased eyes.

Our aim of this study was to characterize the cytokine secretion patterns and proliferative capacities of CD4^+^ and CD8^+^ T cells after subretinal injection of immunodominant peptides [Ubiquitously Transcribed tetratricopeptide repeat gene Y-linked (UTY) and DEAD Box polypeptide 3 Y-linked (DBY)] of the HY male antigen, in C57BL/6 female mice. Because UTY is restricted to MHC-I and DBY to MHC-II, they enable the specific characterization of CD4^+^ (Th) and CD8^+^ (Tc) T cells. We showed that subretinal injection of HY peptides followed by subcutaneous HY immunization did not induce immune deviation, but rather global systemic T-cell inhibition specific for the injected antigen.

## Materials and Methods

### Animals

Wild-type 6- to 8-week-old C57BL/6 female mice (H-2^b^) were purchased from Charles River Laboratories (L’Arbresle, France). Animals were anesthetized either by intraperitoneal injection of 120 mg/kg ketamine (Virbac, Carros, France) and 6 mg/kg xylazine (Bayer, Lyon, France) or by inhalation of isoflurane (Baxter, Guyancourt, France). Mice were euthanized by cervical elongation. All mice were housed, cared for, and handled in accordance with the ARVO Statement for the Use of Animals in Ophthalmic and Vision Research, European Union guidelines, and with the approval of the local research ethics committee (CEEA-51 Ethics Committee in Animal Experimentation, Evry, France; Authorization number APAFIS#2388-2015102117539948).

### Peptides

The DBY and UTY peptides, NAGFNSNRANSSRSS, and WMHHNMDLI, respectively, were synthesized by Genepep (Montpellier, France) and shown to be more than 95% pure. Ovalbumin was purchased from Invivogen (Toulouse, France).

### Subretinal Injections

The eye was protruded under microscopic visualization and perforated with a 27 G beveled needle. A blunt 32 G needle set on a 10 µL Hamilton syringe was inserted in the hole and 2 µL of PBS or UTY + DBY was injected into the SR. The quality of the injection was verified by checking the detachment of the retina.

### Subcutaneous Injections

PBS or UTY + DBY were emulsified in Complete Freund’s Adjuvant (Sigma, Lyon, France) at a 1:1 ratio and 100 µL of the preparation (200 µg of UTY + DBY/mouse) was injected at the base of the tail.

### Cell Extraction from Spleen and Inguinal Lymph Nodes

After euthanasia, spleens were removed and crushed with a syringe plunger on a 70-µm filter in 2 mL of RPMI medium. Red cells were lysed by adding ACK buffer (8.29 g/L NH4Cl, 0.037 g/L EDTA, and 1 g/L KHCO3) for 1 min. Lysis was stopped by addition of complete RPMI medium (10% FBS, 1% penicillin/streptomycin, 1% glutamine, and 50 µM β-mercaptoethanol). After centrifugation, cells were counted and the concentration was adjusted in complete RPMI medium.

Inguinal lymph nodes were crushed with a syringe plunger in 2 mL of RPMI medium. Debris was eliminated by transferring the supernatants into new tubes. After centrifugation, cells were counted and the concentration was adjusted in complete RPMI medium.

### ELISpot Assay

IFN-γ Enzyme-Linked Immunospot plates (MAHAS45, Millipore, Molsheim, France) were coated with anti-IFNγ antibody (eBiosciences, San Diego, CA, USA) overnight at +4°C. Stimulation media (complete RPMI), UTY (2 µg/mL), DBY (2 µg/mL), UTY + DBY (2 µg/mL), or Concanavalin A (Sigma, Lyon, France) (5 µg/mL) were plated and 5.10^5^ splenocytes/well were added. After 24 h of culture at +37°C, plates were washed and the secretion of IFNγ was revealed with a biotinylated anti-IFNγ antibody (eBiosciences), Streptavidin-Alcalin Phosphatase (Roche Diagnostics, Mannheim, Germany), and BCIP/NBT (Mabtech, Les Ulis, France). Spots were counted with an AID ELISpot Reader system ILF05 and the AID ELISpot Reader v6.0 software.

### Proliferation Assay

Stimulation media [complete RPMI, UTY (2 µg/mL), DBY (2 µg/mL), UTY + DBY (2 µg/mL) or Concanavalin A (5 µg/mL)] were plated and 10^5^ cells from inguinal lymph nodes were added per well. After 48 h of culture at +37°C, 1 μCi of ^3^H-Thymidine (Perkin, Villebon, France) was added to each well. After 18 h of culture at +37°C, DNA was harvested and radioactive incorporation was measured by standard liquid scintillation counting. Results are expressed as counts per minute.

### Cytokine Titration by Cytometric Bead Array

Stimulation media [medium, UTY (2 µg/mL), DBY (2 µg/mL), UTY + DBY (2 µg/mL), or Concanavalin A (5 µg/mL)] were plated and 10^6^ splenocytes/well were added. After 36 h of culture at +37°C, supernatants from triplicates were pooled and frozen at −80°C until the titration. Cytometric bead arrays were performed with BD Biosciences flex kits (IL-2, IL-4, IL-6, IL-10, IL-13, IL-17A, IFN-γ, GM-CSF, TNFα, and MCP-1). Briefly, capture bead populations with distinct fluorescence intensities and coated with cytokine-specific capture antibodies were mixed together. Next, 25 µL of the bead mix of beads was distributed and 25 µL of each sample (supernatants) was added. After 1 h of incubation at room temperature, cytokine-specific PE-antibodies were mixed together and 25 µL of this mix was added. After 1 h of incubation at room temperature, beads were washed with 1 mL of Wash buffer and data were acquired with an LSRII flow cytometer (BD Biosciences). FCAP software (BD Biosciences) was used for the analysis.

### Statistical Analysis

Statistical analyses were performed with GraphPad Prism V6.0. After ANOVA, Tukey’s test was performed. *P*-value < 0.05, *; <0.01, **; <0.001, ***; <0.0001, ****. Principal component analysis was performed by a biostatistician with R software.

## Results

### Inhibition of IFNγ From the Th1 Profile by Subretinal Injection of HY Peptides

**Figure 1:**
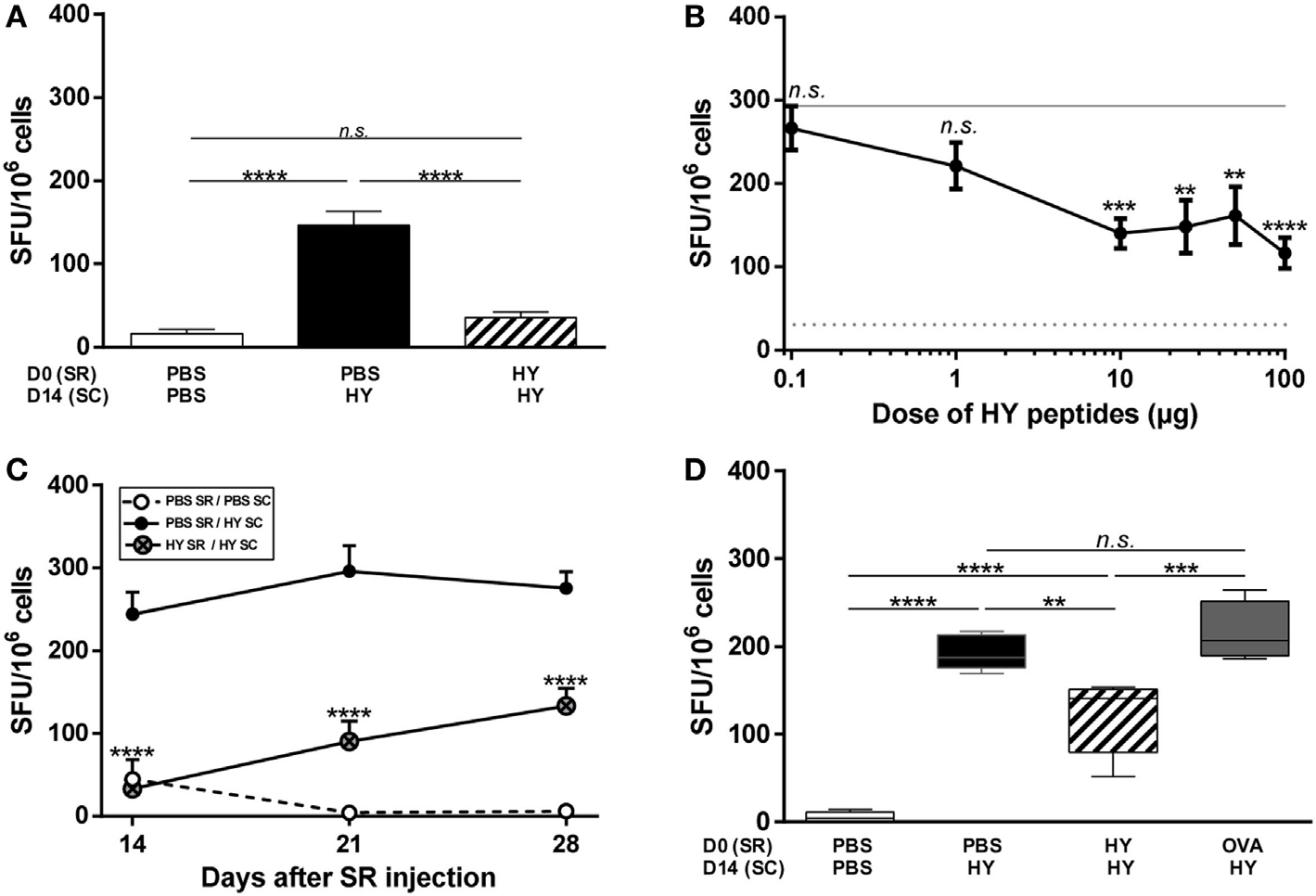
Inhibition of the T-cell immune response after subretinal injection of HY peptides. **(A)** PBS or 50 µg of UTY + DBY (HY) peptides were injected in the subretinal space (SR) of mice on day 0. 2 weeks later, the immune response was challenged by subcutaneous immunization (SC) of either PBS:CFA or HY:CFA (The number *N* of experiments is 6). **(A–D)** The immune response of total splenocytes re-stimulated *in vitro* by HY peptides was assessed 1 week after immunization by IFNγ ELISpot (5 mice/group/experiment). **(B)** PBS or different doses of HY peptides (0–100 µg) were injected in the SR of mice on day 0. 2 weeks later, the immune response was challenged by a subcutaneous immunization of either PBS:CFA or HY:CFA, and the analysis was performed 1 week after the immunization (*N* = 3). **(C)** PBS or 50 µg of HY peptides was injected in the SR of mice on day 0. 1, 2, or 3 weeks later, the immune response was challenged by a subcutaneous immunization of either PBS:CFA (PBS SR/PBS SC group) or HY:CFA (PBS SR/HY SC group; HY SR/HY SC group) (*N* = 2). **(D)** PBS, HY peptides, or ovalbumin were injected in the SR of mice on day 0. 2 weeks later, the immune response was challenged by a subcutaneous immunization of either PBS:CFA or HY:CFA (Data representative from 1 of 2 experiments). All results are represented as mean ± SEM. Statistical analysis: ANOVA, Tukey post-test. **(B,C)** Statistics presented are the results of HY SR/HY SC group compared to PBS SR/HY SC group. SFU, spot forming units.

Anterior chamber-associated immune deviation is described as inhibition of the Th1 profile, revealed by DTH experiments and ELISpot assays (the reference method for measuring IFNγ secretion). To determine whether the pattern of inhibition in the SR is similar to that of ACAID, mice received either PBS or HY peptides (UTY + DBY) in the SR on day 0, and were immunized subcutaneously with the HY peptides 2 weeks later to challenge the immune response. Spleen cells were harvested on day 21 and stimulated *in vitro* with the HY peptides to quantify IFNγ secretion by HY-specific T cells with ELISpot. As a negative control, a group of mice received PBS in the SR on day 0 and PBS subcutaneously on day 14 (Figure 1A; Figure S1 in Supplementary Material); as expected, very few spleen cells from this group secreted IFNγ in response to HY peptides 1 week later. Conversely, a positive control group of mice received PBS in the SR on day 0 and the HY peptides subcutaneously on day 14. In this case, 150–250 spot forming units (SFUs) were counted in response to HY peptides, corresponding to IFNγ-secreting spleen cells. The number of SFUs from the experimental group of mice receiving the HY peptides in the SR before immunization with the same peptides was significantly lower than in the positive controls (*p*-value < 0,0001; 75.5% inhibition) and similar to that by the negative control group (Figure 1A). To better characterize this phenomenon, the HY doses for the subretinal injections ranged from 0.1 to 100 µg of HY peptides. When 0.1 or 1 µg of HY peptides was injected in the SR, the level of IFNγ secretion by T cells was similar to that in the positive control group. At doses of 10–100 µg, however, the level of IFNγ secretion by T cells was significantly lower than that in the positive controls (Figure 1B). The limited volume that can be injected in the SR made it impossible to test higher doses.

Next, we assessed the kinetics of the induction of this phenomenon by immunizing mice 1, 2, or 3 weeks after the subretinal injection and analyzed IFNγ secretion by spleen cells 1 week after HY immunization. The level of IFNγ secretion varied with the time point (*p* = 0.0006 between day 14 and day 28 for HY-injected group), but was always significantly inhibited by 50–80% compared with the positive controls (Figure 1C). Finally, to check the antigen-specificity of the inhibition of the Th1 profile, ovalbumin was injected into the SR 2 weeks before immunization with HY peptides. The level of IFNγ secretion by T cells in response to HY peptides in this condition was similar to that for the positive controls and thus indicated that OVA injection does not induce inhibition of the immune response to HY peptides (Figure 1D). Although the percentage of inhibition between the positive control and HY-injected mice was not identical to the one in Figure 1A, the inhibition was still significant (*p* = 0.0055) and the variation is likely due to biological heterogeneity. Together, these data show that subretinal injection of HY peptides induced antigen-specific inhibition of IFNγ secretion (characteristic of a Th1/Tc1 profile) by HY-specific T cells.

### Inhibition of CD4^+^ T Cell Proliferation by Subretinal Injection of HY Peptides

**Figure 2:**
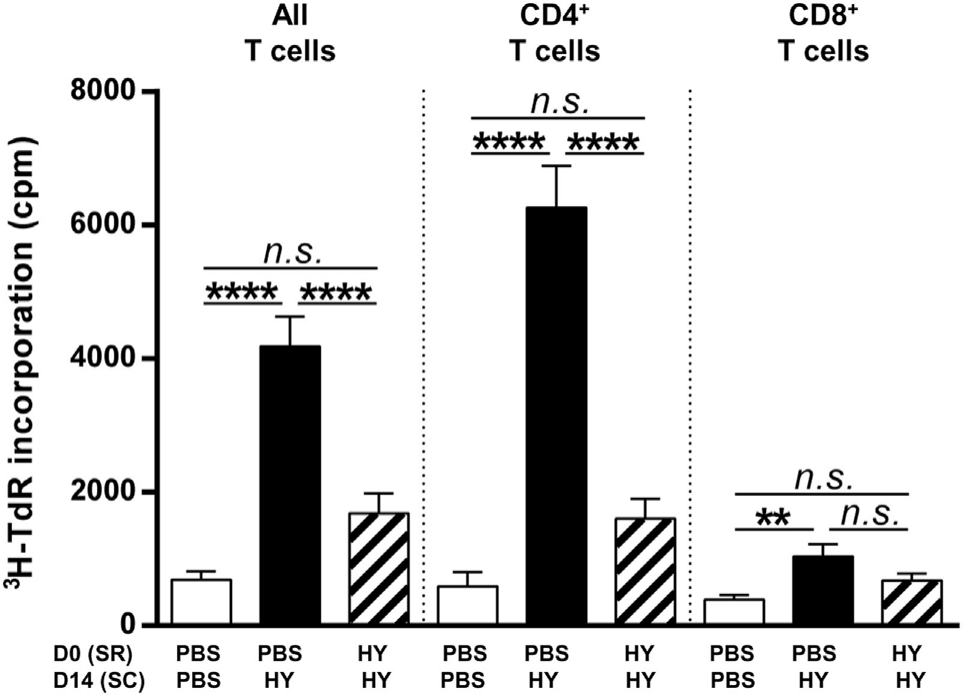
Clonal expansion of T cells. PBS or 50 µg of Ubiquitously Transcribed tetratricopeptide repeat gene Y-linked (UTY) + DEAD Box polypeptide 3 Y-linked (DBY) (HY) peptides were injected in the subretinal space (SR) of mice on day 0. 2 weeks later, the immune response was challenged by a subcutaneous immunization (SC) of either PBS:CFA or HY:CFA (*N* = 7). 1 week after immunization, the proliferative capacity of T cells extracted from inguinal lymph nodes, *in vitro* re-stimulated by UTY, DBY, or UTY + DBY peptides (5 mice/group/experiment) was assessed. The results are represented as mean ± SEM. Statistical analysis: ANOVA, Tukey post-test.

To characterize in greater depth how the subretinal injection of HY peptides affected peripheral T cells, cells from inguinal lymph nodes (draining the immunization site) were stimulated *in vitro* with UTY + DBY (all T cells), DBY alone (CD4^+^ T cells), or UTY alone (CD8^+^ T cells) for a proliferation assay. A test of radiolabeled thymidine incorporation showed that UTY + DBY *in vitro* stimulation, which activates both CD4^+^ and CD8^+^ T cells, inhibited this incorporation by more than 50% in mice that received HY peptides in the SR before immunization compared to control mice receiving PBS. This result thus shows that the subretinal injection of HY peptides induced inhibition of HY-specific T-cell proliferation. Specific sources of stimulation were used to investigate the subclass of T cells involved. Under DBY stimulation, we observed that inhibition of the ^3^H-TdR incorporation into CD4^+^ T cells from mice with subretinally injected HY-peptides was not significantly different to that in the negative control group and 75% less than in the positive control. Under UTY stimulation, however, despite poor incorporation of radiolabeled thymidine by CD8^+^ T cells in the positive group, proliferation of these cells did not differ significantly from that observed in the experimental group (the mice with subretinal injections of HY peptides) (Figure 2). Alternative methods may be better suited than proliferation assays to evaluate the impact of the subretinal peptide injections on CD8^+^ T cell responses. Taken together, these results provide evidence that the subretinal injection of HY peptides mainly inhibited the proliferation of HY-specific CD4^+^ T cells.

### Global Inhibition of T-Cell Polarization by Subretinal HY-Peptide Injection

**Figure 3:**
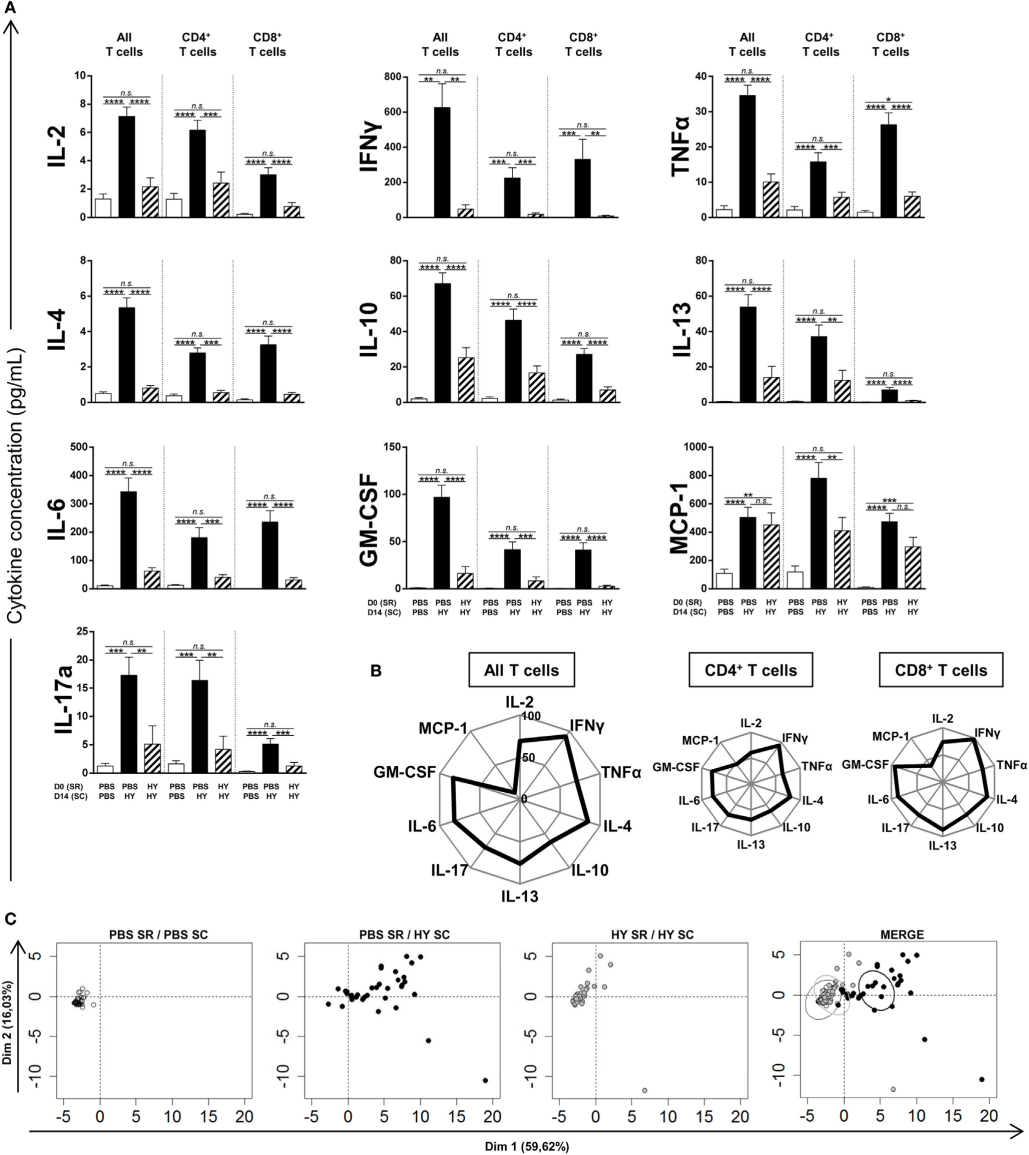
Titration of cytokines secreted by T cells. PBS or 50 µg of Ubiquitously Transcribed tetratricopeptide repeat gene Y-linked (UTY) + DEAD Box polypeptide 3 Y-linked (DBY) HY peptides were injected in the subretinal space (SR) of mice at day 0. 2 weeks later, the immune response was challenged by a subcutaneous immunization (SC) of either PBS:CFA or HY:CFA (*N* = 4–7). 1 week after the immunization, total splenocytes were re-stimulated *in vitro* by UTY, DBY, or UTY + DBY peptides. After 36 h of culture, supernatants were removed and titrated for the indicated cytokines (5 mice/group/experiment). **(A)** The results are represented as mean ± SEM. Statistical analysis: ANOVA, Tukey post-test. **(B)** Kiviat diagram representing the percentage of inhibition of secretion in the HY SR/HY SC group compared to the PBS SR/HY SC group. **(C)** Principal component analysis of data presented in panel **(A)**. Each point represents an individual mouse. On the merged presentation (last panel), the ellipses represent the 95% confidence interval of a bivariate normal distribution.

After the proof of concept that subretinal injection of the HY peptides can induce HY-specific inhibition of IFNγ secretion (from the Th1 profile) and T-cell proliferation, we investigated the effect of this injection on other secretion profiles. Spleen cells were cultured for 40 h under conditions of *in vitro* stimulation by UTY + DBY, or DBY, or UTY. Characteristic cytokines of Th1/Tc1, Th2/Tc2, Th17/Tc17, inflammation, and chemoattraction were then measured in the culture supernatant to determine the polarization of these cells (Figure 3A). Spleen cells from the experimental group of mice subretinally injected with HY peptides secreted significantly lower amounts of cytokines, apart from MCP-1, than those from the positive controls, and in a profile similar to that of the negative control. Chemoattractive MCP-1 secretion did not differ from the positive group in the CD8^+^ T cells, but was significantly lower in the CD4^+^ T cells. By calculating the percentage of inhibition of the secretion of each cytokine compared to the positive control (Figure 3B), we observed that secretion of Th1/Tc1, Th2/Tc2, and Th17/Tc17, and inflammatory cytokines was inhibited by 75% to almost 100% in CD4^+^ and CD8^+^ T cells compared to the positive control. To provide a global, integrated view of this cytokine signature, values of secretions by CD4^+^, CD8^+^, and all T cells for each mouse were used to perform a principal component analysis (Figure 3C). Dimension 1 and dimension 2 distribute mice along the *X* and the *Y*-axis, respectively, and account for almost 60 and 16% of the differences between mice. Mice that received PBS in the SR were subcutaneously injected with PBS at day 14, from the negative control group, are clustered around the origin of the axis. Mice from the positive control group, which received PBS in the SR and were immunized with HY peptides, are distributed along the *X* and *Y*-axis. Mice from the experimental group, which received HY peptides in the SR before HY immunization are clustered around the origin of the axis. The final figure merges these PCA results, with ellipses showing the 95% confidence interval of the cytokine secretion patterns of each group: similar for the mice from the negative control group and from the experimental group, and different for those in the positive control group. Altogether, these data show that the subretinal injection of HY peptides induced a global HY-specific inhibition of Th1/Tc1, Th2/Tc2, Th17/Tc17, and inflammatory cytokines.

## Discussion

The anterior chamber of the eye is known for its induction of antigen-specific peripheral modulation of the immune response after antigen injection, a characteristic that has been considered unique to this organ. Recent studies have begun to reveal additional immunomodulatory characteristics of the SR, showing that this route of injection can also inhibit immune response (11). However, although the proof-of-principle is widely accepted, no broad characterization of cytokine and proliferative capacities of T cells have been performed yet. This study seeks to provide an in-depth characterization of the impact of antigen injection into the SR on the immune response and to decipher the respective contributions of CD4^+^ and CD8^+^ T cells.

Our results clearly show that the subretinal injection of peptides induces an antigen-specific inhibition of CD4^+^ T-cell proliferation and a global inhibition of proinflammatory cytokines, more specifically Th1/Tc1, Th2/Tc2, and Th17/Tc17 profiles. Our results thus appear to be in accordance with those of Nakamura et al. in 2005: they showed in an ACAID model that this mechanism does not cause a deviation from a Th1 to a Th2 profile, but instead inhibits the Th1 as well as the Th2 profile (9). Thus, this mechanism does not involve the antagonism between Th1 and Th2 profiles, but can present a quite classical pattern of inhibition of antigen-specific T cells. To go further in deciphering the underlying mechanisms, it would be interesting to study the involvement of HY-specific regulatory T cells after subretinal injection of HY peptides in further investigations. McPherson et al. reported the generation of natural and peripheral Tregs in a model of neo-self retinal protein expression, and also reported the possibility to induce local Tregs in this model (12, 13). However, no study was performed in the context of a subretinal injection and similar experiments should be achieved to pinpoint the mechanisms underlying the immune modulation that we have observed. Is this mechanism due to an active suppression by Tregs or is it mediated by a deletion of antigen-specific conventional T cells? Moreover, our results showed that the inhibition of T cells induced by the subretinal injection of peptides is weaker at day 28 compared to day 14, which questions the transient nature of the mechanism. Thus, other experiments should seek to characterize the long-term kinetics of SRAII to understand if the effect is transient or can be maintained over time. In a different context, Yamada et al. have shown that murine primary corneal endothelial cells from C57BL/6 mice injected in the anterior chamber of BALB/c mice induce tolerance for as long as 8 weeks after injection (16). To our knowledge, however, the long-term immune consequences of subretinal injection have not been studied, yet despite the special interest in the context of subretinal gene therapy, which induces the expression of a protein (e.g., RPE65) over a long period of time (15, 17, 18).

The multiple similarities between the immune inhibition observed after the subretinal injection of antigen and ACAID lead us to propose an acronym for this phenomenon: SRAII, for subretinal-associated immune inhibition. This inhibitory mechanism may be of interest to understand or to modulate immune responses in different therapeutic approaches. The immune privileges of the eye and tolerogenic antigen presenting cells have been proposed to be useful in a wide range of pathologies (19). The SR, despite the complexity of gaining access to it, displays strong immune modulatory properties that may be exploited to control systemic inflammation. Future studies seem to be warranted to determine if SRAII can be induced in the context of inflammatory pathologies in the eye.

## Ethics Statement

All mice were housed, cared for, and handled in accordance with the ARVO Statement for the Use of Animals in Ophthalmic and Vision Research, European Union guidelines, and with the approval of the local research ethics committee (CEEA-51 Ethics Committee in Animal Experimentation, Evry, France; Authorization number APAFIS#2388-2015102117539948).

## Author Contributions

JV designed the experiments, performed experiments, analyzed data, and wrote the manuscript. SD and QK performed experiments. AG contributed to critical revision of the manuscript. SF designed the experiments, analyzed data, wrote the manuscript, and revised it.

## Conflict of Interest Statement

The authors declare that the research was conducted in the absence of any commercial or financial relationships that could be construed as a potential conflict of interest.

## Supplementary Material

Figure S1 Reprensentative ELISpots of data presented in Figure 1A. PBS or 50 µg of Ubiquitously Transcribed tetratricopeptide repeat gene Y-linked + DEAD Box polypeptide 3 Y-linked (HY) peptides were injected in the subretinal space (SR) of mice on day 0. 2 weeks later, the immune response was challenged by subcutaneous immunization (SC) of either PBS:CFA or HY:CFA. The immune response of total splenocytes re-stimulated *in vitro* by HY peptides was assessed 1 week after immunization by IFNγ ELISpot. We also performed controls using cells cultured in complete RPMI medium (No stimulation) or cultured with Concanavalin A.

## Abbreviations

ACAID, anterior chamber-associated immune deviation; DBY, DEAD Box polypeptide 3 Y-linked; DTH, delayed-type hypersensitivity; OVA, ovalbumin; SFU, spot forming unit; SRAII, subretinal-associated immune inhibition; UTY, Ubiquitously Transcribed tetratricopeptide repeat gene Y-linked.
