# Supplementary figures and images for: Subretinal Injection of HY Peptides Induces Systemic Antigen-Specific Inhibition of Effector CD4+ and CD8+ T-Cell Responses
Source: Front Immunol. 2018 Mar 13;9:504. doi: 10.3389/fimmu.2018.00504 (PMC5890180; doi:10.3389/fimmu.2018.00504)

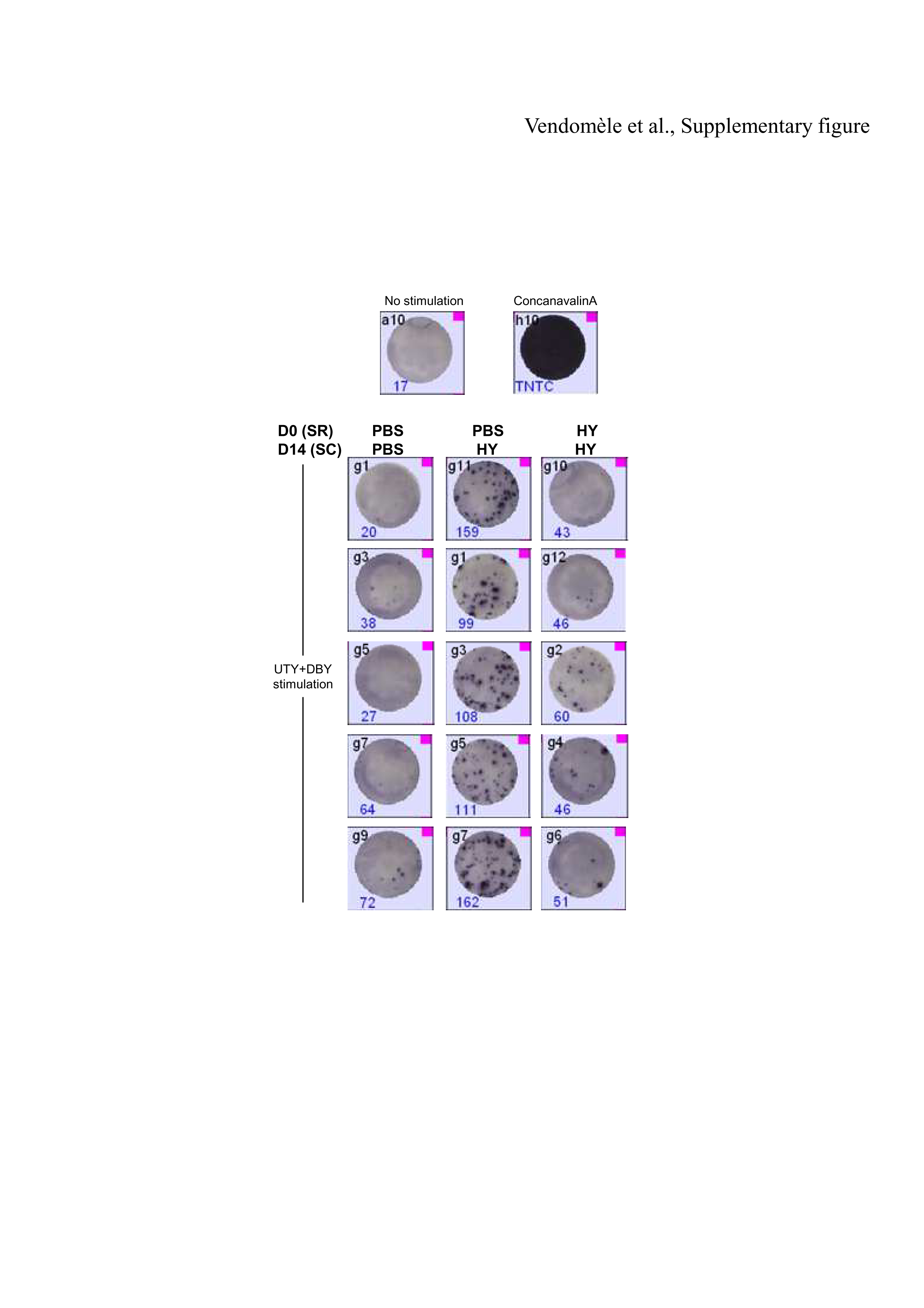

Supplement: Figure S1 — Reprensentative ELISpots of data presented in Figure 1A. PBS or 50 µg of Ubiquitously Transcribed tetratricopeptide repeat gene Y-linked + DEAD Box polypeptide 3 Y-linked (HY) peptides were injected in the subretinal space (SR) of mice on day 0. 2 weeks later, the immune response was challenged by subcutaneous immunization (SC) of either PBS:CFA or HY:CFA. The immune response of total splenocytes re-stimulated in vitro by HY peptides was assessed 1 week after immunization by IFNγ ELISpot. We also performed controls using cells cultured in complete RPMI medium (No stimulation) or cultured with Concanavalin A. [file image_1.TIF]
